# Specialized stellate cells offer a privileged route for rapid water flux in *Drosophila* renal tubule

**DOI:** 10.1073/pnas.1915943117

**Published:** 2020-01-06

**Authors:** Pablo Cabrero, Selim Terhzaz, Anthony J. Dornan, Saurav Ghimire, Heather L. Holmes, Daniel R. Turin, Michael F. Romero, Shireen A. Davies, Julian A. T. Dow

**Affiliations:** ^a^Institute of Molecular, Cell and Systems Biology, College of Medical, Veterinary and Life Sciences, University of Glasgow, G12 8QQ Glasgow, United Kingdom;; ^b^Department of Physiology and Biomedical Engineering, Mayo Clinic College of Medicine & Science, Rochester, MN 55905;; ^c^Division of Nephrology and Hypertension, Mayo Clinic College of Medicine & Science, Rochester, MN 55905;; ^d^University of Minnesota Rochester, Rochester, MN 55905

**Keywords:** Malpighian tubule, *Drosophila melanogaster*, aquaporin, *Xenopus* oocyte, stellate cell

## Abstract

Insects are highly successful, in part through an excellent ability to osmoregulate. The renal (Malpighian) tubules can secrete fluid faster on a per-cell basis than any other epithelium, but the route for these remarkable water fluxes has not been established. In *Drosophila melanogaster*, we show that 4 genes of the major intrinsic protein family are expressed at a very high level in the fly renal tissue: the aquaporins (AQPs) *Drip* and *Prip* and the aquaglyceroporins *Eglp2* and *Eglp4*. As predicted from their structure, and by their transport function by expressing these proteins in *Xenopus* oocytes, Drip, Prip, and Eglp2 show significant and specific water permeability, whereas Eglp2 and Eglp4 show very high permeability to glycerol and urea. Knockdowns of any of these genes result in impaired hormone-induced fluid secretion. The *Drosophila* tubule has 2 main secretory cell types: active cation-transporting principal cells, wherein the aquaglyceroporins localize to opposite plasma membranes, and small stellate cells, the site of the chloride shunt conductance, with these AQPs localizing to opposite plasma membranes. This suggests a model in which osmotically obliged water flows through the stellate cells. Consistent with this model, fluorescently labeled dextran, an in vivo marker of membrane water permeability, is trapped in the basal infoldings of the stellate cells after kinin diuretic peptide stimulation, confirming that these cells provide the major route for transepithelial water flux. The spatial segregation of these components of epithelial water transport may help to explain the unique success of the higher insects in regulating their internal environments.

## Significance

The tiny insect renal (Malpighian) tubule can transport fluid at unparalleled speed, suggesting unique specializations. Here we show that strategic allocation of major intrinsic proteins (MIPs) to specific cells within the tubule allows the separation of metabolically intense active cation transport from passive chloride and water conductance. This specialized renal architecture is general to at least many higher insects, providing a clue to the unique success of the class Insecta in colonizing an extraordinary range of ecological niches.

There are more species of insects than all other forms of life combined. In part, this is because of the exceptional ability of the simple body plan to operate in a wide range of environments, and osmoregulation is a key component of this success. Remarkably, the insect Malpighian (renal) tubule is capable of secreting fluid faster (on a per cell volume basis) than any other epithelium known (1, 2), and shows an extremely high osmotic water permeability (*P*_osm_) (3). In *Drosophila*, the renal tubule has 2 major cell types (4–6); the mitochondria-rich principal cell actively transports protons via an apical, plasma membrane vacuolar H^+^-ATPase (V-ATPase) (7), setting up a gradient which is exchanged primarily for potassium (8, 9), which enters the cell basolaterally through the combined activity of Na^+^, K^+^-ATPase (10), inward rectifier potassium channels (11–13) and potassium cotransports (14–16). The smaller stellate cell (17, 18) provides a route for hormone-stimulated (19–22) chloride conductance through a basolateral ClC-a chloride channel (23), partnered with secCl, an apical cys-loop chloride channel (24), to balance the lumen-positive charge, and so effect a net movement of salt. Aquaporins (AQPs; the water transporting major intrinsic proteins [MIPs]) are known to be highly expressed in insect tubules (25–29), and global knockdown of an AQP in the *Aedes* mosquito (30–32), or in the beetle *Tribolium* (33), impacts water loss. Although in situ hybridization of *Drip* showed expression in stellate cells (25), the route or mechanism of the very high osmotically obliged water fluxes that produce such remarkable fluid output has not been characterized. Here, using the powerful cell-specific transgenic technologies unique to *Drosophila melanogaster* (34), we show that this flux is transcellular, and, selectively through the stellate cells, mediated by 2 AQPs, in response to diuretic hormone stimulation. Knockdown of AQPs in stellate cells impacts survival under stress, and comparative studies suggest that water flux is confined to specific cell types in tubules from a broad phylogenetic range of insects.

## Results and Discussion

### Tubules Express 4 Members of the MIP Family.

**Fig. 1.**
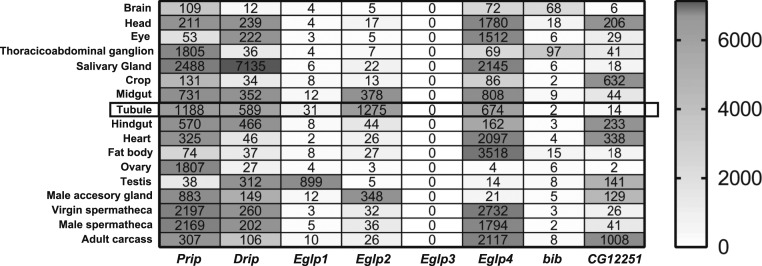
MIP family expression in *Drosophila melanogaster*. Data mining of FlyAtlas.org identified 4 MIP genes (*Prip*, *Drip*, *Eglp2*, and *Eglp4*) with highly abundant expression in adult Malpighian tubules. FlyAtlas expression levels are derived from normalized Affymetrix microarray data, and are shaded according to the scale on the right.

MIPs are a multigene family of 6-transmembrane domain proteins that assemble as tetramers to form pores (35). Most members of the family are true water channels (AQPs); others can facilitate movement of water or small organic molecules (aquaglyceroporins); but the substrates of some are still obscure (35). In *Drosophila*, 8 genes make up the MIP family (Fig. 1), but the FlyAtlas and FlyAtlas2 gene expression online resources (27, 36, 37) independently report that only 4 are expressed at high levels in epithelia such as the salivary gland, midgut, hindgut, and Malpighian tubules (Fig. 1). Two of these highly expressed genes (*Drip* and *Prip*) are similar to classical AQPs in structure, whereas the other 2 (*Eglp2* and *Eglp4*) align with the aquaglyceroporins (38). Comparison of the protein sequence of *D. melanogaster* AQPs (Drip and Prip) and aquaglyceroprins (Eglp2 and Eglp4) in a Clustal Omega alignment shows that key active-site residues, including those required for water selectivity and those involved for their regulation, have been conserved (*SI Appendix*, Fig. S1). There are thus at least 2 candidates that could mediate high water flux rates in polarized epithelia.

### Each MIP Localizes to a Different Membrane Domain within the Tubule.

**Fig. 2.**
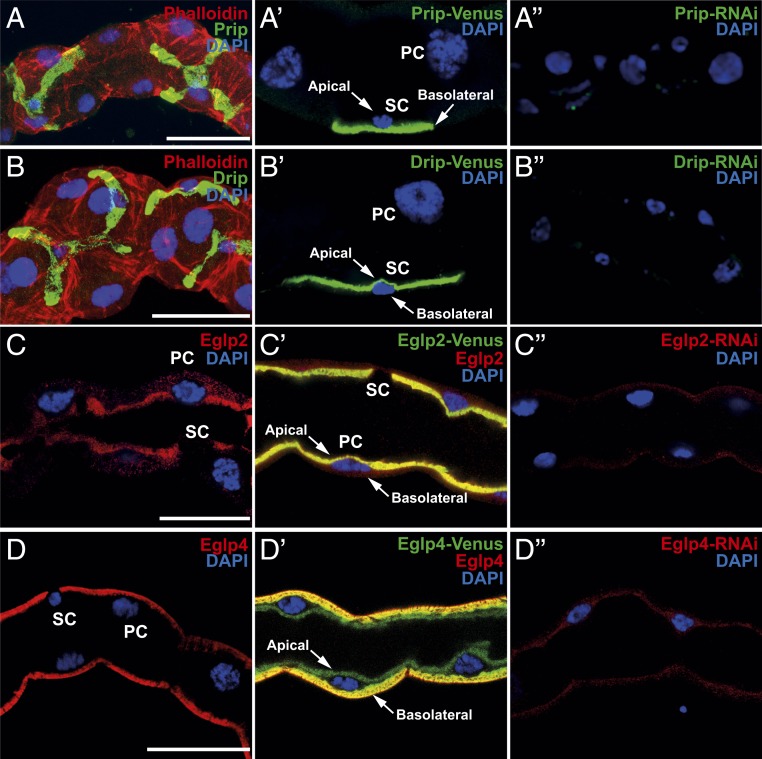
Subcellular localization of MIPs in the *Drosophila* tubule. (*A* and *B*) Prip and Drip localize to opposite plasma membranes of the stellate cell (SC). (*A*) Prip is expressed in basolateral membrane (green) and is in contact with the outside of the tubule, or hemolymph and (*B*) Drip in apical membrane (green) and faces the lumen of the tubule. DAPI (blue) staining for nuclei and phalloidin (red) staining for actin are shown. Blue (DAPI) nuclei are shown to allow distinction between basal and apical distribution of MIPs, emphasized with arrows. (*A′* and *B′*) Overexpression of Prip and Drip labeled with Venus (eYFP) recapitulate the pattern of expression observed by immunocytochemistry. (*C* and *D*) Eglp2 and Eglp4 localize to opposite, plasma membranes of the principal cell (PC). (*C*) Eglp2 is expressed in apical membrane (red) and (*D*) Eglp4 is expressed in basolateral membrane (red), and DAPI (blue). (*C′* and *D′*) Colocalization (yellow) (*C′*) between Eglp2-Venus and Eglp2 to the apical membrane and (*D′*) between Eglp4-Venus and Eglp4 to the basolateral membrane. (*A″*–*D″*) Down-regulation of MIPs in specific cell types using RNAi reduces protein levels. (Scale bar, 40 µm.)

Water and solutes transport is achieved by an apicobasally polarized distribution of membrane proteins, and, accordingly, it is important to establish where in the tubule principal and stellate cells MIPs reside. We raised specific antibodies against the 4 tubule-expressed MIPs and validated them by Western blotting (*SI Appendix*, Figs. S2 and S3). Immunocytochemistry showed clear segregation of MIP expression, with the 2 AQPs expressed on opposite sides of the specialized stellate cell (Drip and Prip are localized to the apical and basolateral membranes, respectively; Fig. 2 *A* and *B*), and the 2 aquaglyceroporins expressed on opposite sides of the main principal cell (Eglp2 and Eglp4 are localized to the apical and basolateral membranes, respectively; Fig. 2 *C* and *D*). Accordingly, overexpression of all 4 MIPs labeled with Venus (eYFP) recapitulate the pattern of expression observed by immunocytochemistry (Fig. 2 *A′*–*D*′). These data are consistent with other reports that Drip and Prip show spatial separation in other insects, such as silkworm (39). It would thus be tempting to surmise that the stellate cell provides a major route for water flux through the tissue, but only the transport properties of one of these MIPs (Drip) has been established (25); how many of them are, in fact, functional AQPs?

### Stellate Cell MIPs Are AQPs; Principal Cell MIPs Are Aquaglyceroporins.

**Fig. 3.**
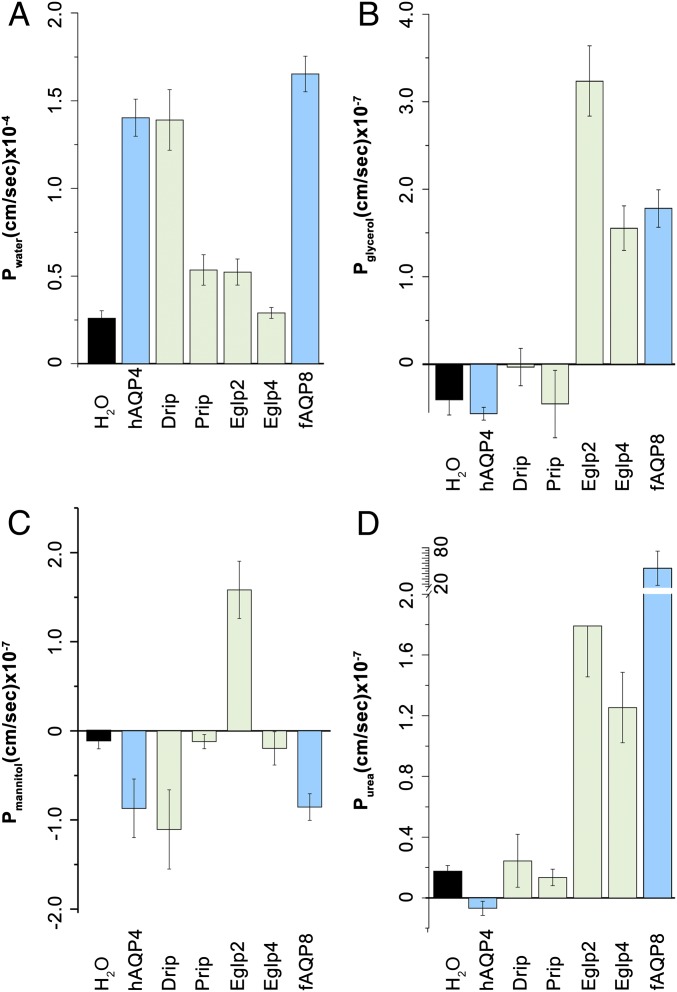
Transport specificity of *Drosophila* tubule-enriched MIPs. Water-injected control oocytes or oocytes expressing *Drosophila* MIPs (Drip, Prip, Eglp2, and Eglp4), human AQP4 (hAQP4, a control classical AQP), and mefugu AQP8 (fAQP8, a control aquaglyceroporin) were tested for permeability of (*A*) water (Pf, P_H2O_), (*B*) urea (P_urea_), (*C*) glycerol (P_glycerol_), and (*D*) mannitol (P_mannitol_).

Each of the 4 candidate genes was expressed in *Xenopus* oocytes, and tested both for classical swelling under hypoosmotic stress and for facilitated flux of organic solutes. The 2 channels expressed in tubules (Drip and Prip) both acted as classical AQPs, showing rapid water fluxes but only barely detectable fluxes of organic solutes (Fig. 3 *A* and *B*). By contrast, the Eglp2 channel showed fluxes of water comparable to Prip, but also very rapid fluxes of small organic solutes, such as glycerol and urea; Eglp4 did not permit water flux, but showed extremely rapid flux of organic solutes. These fluxes are consistent with the predicted classification of Eglp2 and Eglp4 as aquaglyceroporins (31). These data are thus in agreement with Drip and Prip providing a transcellular route for water through the stellate cells, and, as the tubule provides a range of physiological readouts (40, 41), this prediction can be tested experimentally.

### Knockdown of AQPs Reduces Fluid Transport and Impacts Survival.

**Fig. 4.**
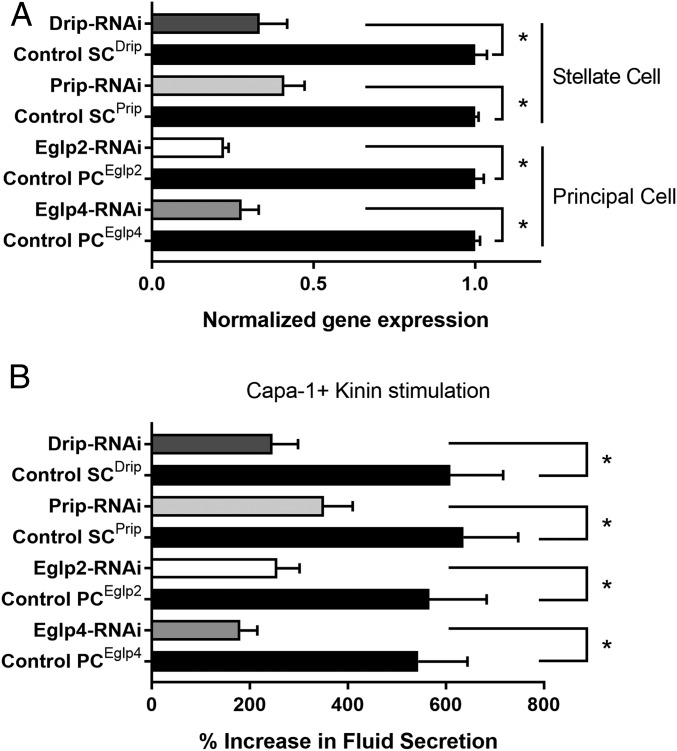
Validation of MIPs knockdowns and impact of cell-specific down-regulation of MIPs on fluid secretion. (*A*) Effects of knockdowns on tubule messenger RNA (mRNA) levels for MIPs, validated by qPCR. Cell-specific down-regulation of *Eglp2* and *Eglp4* in principal cells and *Drip* and *Prip* in stellate cells using their respective UAS-dsRNA lines (protein knockdown achieved by these lines is shown in Fig. 2). Data are expressed as mean fold change compared to parental controls ± SEM (*n* = 3). *P < 0.05 (Student’s *t* test). (*B*) Impact of cell-specific knockdowns of MIPs on stimulated fluid secretion by tubules in response to Capa-1 and Kinin at 10^−7^ M. Data are expressed as percentage increase from basal fluid secretion compared to parental controls ±SEM (*n* = 6 to 10). *P < 0.05 (Student’s *t* test).

Although epithelial polarization of some AQPs has been shown in other insects (25, 28, 39, 42), *Drosophila* genetic technology allows their physiological roles to be dissected with great precision. Using the GAL4/UAS system, which uses the yeast GAL4 transcription factor, a regulator of gene expression of galactose-induced genes, and its recognition site, UAS (Upstream Activating Sequence), and renal cell-type−specific drivers, it is possible to generate transgenic flies in which a single candidate gene is knocked down in only the tubule cell type in which it is expressed, leaving expression throughout the rest of the fly untouched. Accordingly, each of the 4 genes was knocked down in the cell type in which its proteins had been shown to be expressed, and we were able to confirm by qPCR and immunocytochemistry the efficiency of the knockdown of MIPs expression at the gene and protein levels (Figs. 2 *A*′–D′ and 4*A*). The resulting fluid output was then measured under baseline conditions, and when maximally stimulated with diuretic peptides of the capa and kinin families. Knockdown of either *Drip* or *Prip* in just the stellate cell significantly impeded fluid secretion, confirming functional roles in rapid fluid movement across the tissue (Fig. 4*B*). However, knockdown of *Eglp2* or *Eglp4* in the principal cells also elicited reduced fluid secretion rates (Fig. 4*B*). This suggested 2 possibilities: either that all 4 MIPs could produce water conductance, through both cell types (at variance with the biophysical characterization; Fig. 3), or that one pair of channels provided the main route for water, while the other pair allowed flux of a necessary organic osmolyte, or metabolic substrate, such that blockade could reduce overall function of the tissue. In a simple saline containing glucose and glutamine, which has been shown to increase secretion rates in amino acid-free saline (43), similar results were obtained (*SI Appendix*, Fig. S4). The very high rates of generation of primary urine by the tubule could become a liability under dry conditions, and so knockdown of AQPs would be predicted to impact survival under desiccation. This had been shown by global knockdown of the *Drip* ortholog in *Anopheles gambiae*, the malaria vector (28); however, *Drip* is broadly expressed, and so the effect could not be attributed solely to the tubules (30, 31). Using GAL4/UAS technology, we were able to knock down *Drip* or *Prip* expression in just the tubule stellate cells of an otherwise normal insect and show that knockdown of either AQP was sufficient to produce enhanced survival under desiccation stress in female flies (*SI Appendix*, Fig. S5 *A* and *B*). Interestingly, knockdown of either *Eglp2* or *Eglp4* in principal cells did not impact survival to desiccation (*SI Appendix*, Fig. S5 *C* and *D*). Water flux across the tubule is thus limiting for terrestrial insects under desiccation stress, as previously suggested (44), and the reduction in water loss by Malpighian tubules through the stellate cells appears to be an important mechanism for desiccation resistance.

### The Route of Water Flux Is through the Stellate Cells.

**Fig. 5.**
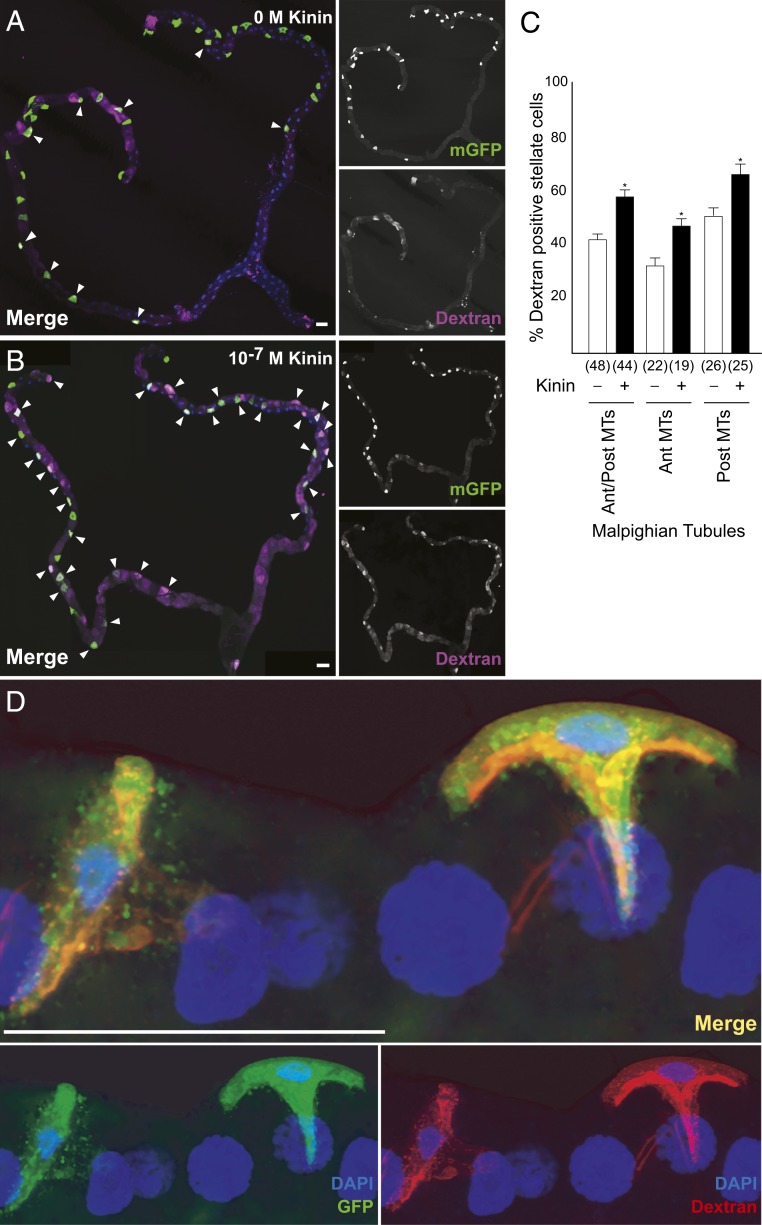
Dextran labeling demonstrates water flux specific to the stellate cells. Accumulation of dextran (*A*) of unstimulated and (*B*) following application of Kinin (10^−7^ M) in tubule expressing mGFP in stellate cells. Because of the length of the tubule, *B* is a photomontage of 3 fields, all captured with the same microscope settings. (*C*) Quantification of dextran labeling. Data are expressed as percentage of dextran-positive stellate cells in response to 10^−7^ M Kinin compared to unstimulated tubule ± SEM (*n* = 44 to 48). *P < 0.05 (Student’s *t* test). (*D*) Maximum Z projection of tubules after application of 40 kDa of dextran conjugated to TRITC dye (red) to tubules in which stellate cells are expressing GFP (green) confirmed the accumulation of dextran to the stellate cell; DAPI, blue. (Scale bars, 50 μm.)

To distinguish the roles of the aquaglyceroporins from the AQPs, it would be necessary to determine the route of water flux through the tubule. The complex polyglucan, dextran, can be readily fluorescently labeled, and can be size-selected to ranges that can be swept along by water flux, but then trapped in a pathway of restricted permeability. Both the principal and stellate cells have apical microvilli, which, in principal cells, are stabilized by the cell adhesion molecule Fasciclin2 (45) and contain mitochondria to support intense activity of the V-ATPase (46); both cell types also possess basal infoldings that increase the available surface area for transport (47). We thus stimulated tubules in the presence of fluorescently labeled 40- to 70-kDa dextran, which pilot experiments had shown was too large to move across the epithelium. Dextran would thus accumulate in a compartment diagnostic of the route of water movement, be it the principal or stellate cells, or the paracellular route between the tight (“septate”) junctions (48). The results showed that only the basal labyrinths of the stellate cells became labeled with 40-kDa dextran (Fig. 5 *A*, *B*, and *D*), and that the percentage of stellate cell population displaying accumulation of dextran was significantly higher after kinin stimulation of fluid secretion (Fig. 5*C*). Although we cannot exclude the possibility, for example, that apical Eglp2 in the principal cells can allow a water flux, perhaps via gap junctions (49, 50) from basolateral Prip in the stellate cells, only the stellate cell has a basolateral functional AQP, and so the pathway provided by Prip and Drip in the stellate cells is likely to be the major route for transepithelial water movement through the tubule.

### Generality of the Stellate Cell Model.

**Fig. 6.**
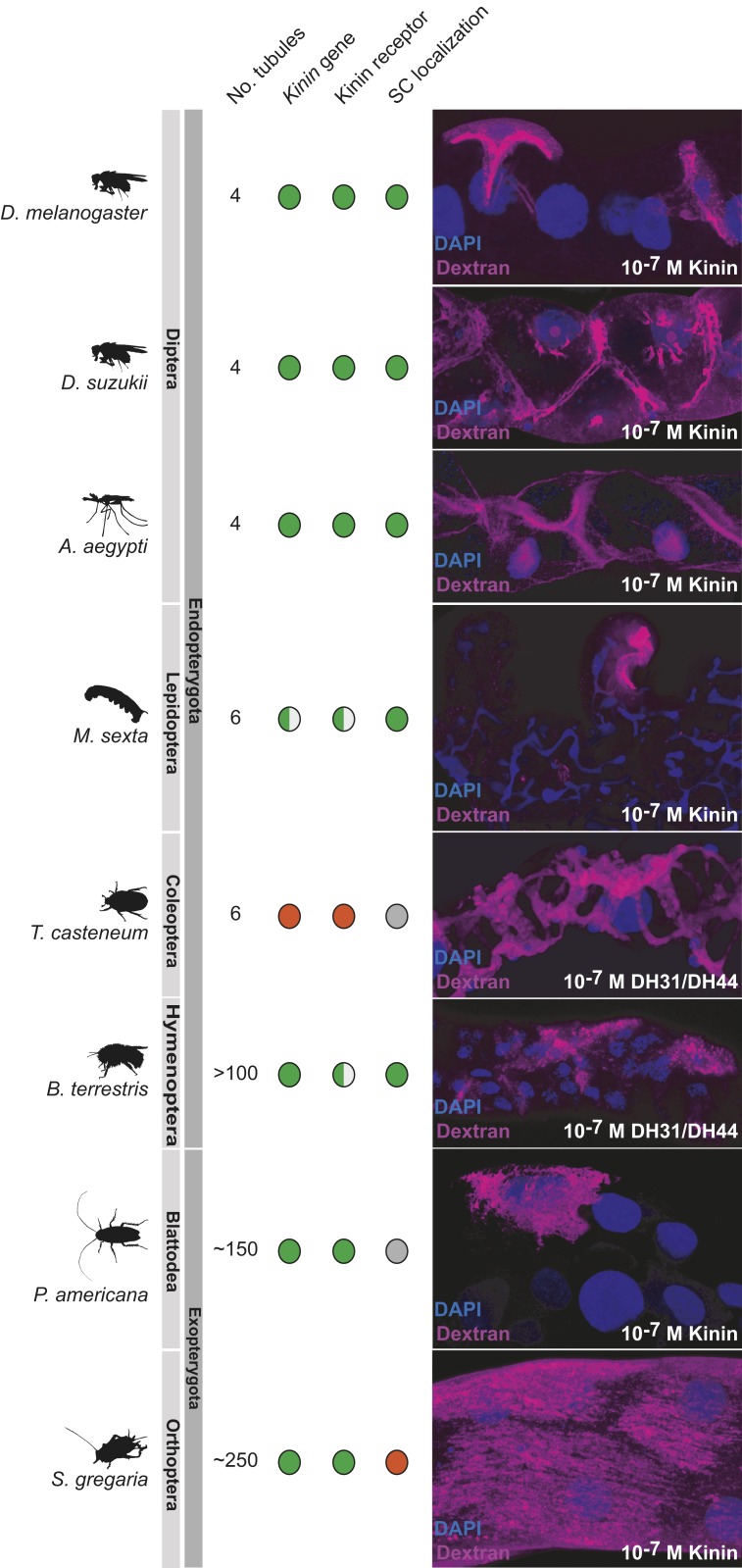
Dextran labeling as a tool to probe insect biodiversity. The dextran labeling protocol employed for *D. melanogaster* was applied to other insect species, selected from the major insect orders of exopterygotes and endopterygotes. The distribution of known stellate cells and Kinin labeling (diagnostic of the route of chloride shunt conductance) is adapted from ref. 51; green denotes positive (gene is present, or kinin receptor localization confined to stellate cells); red denotes absence, and gray denotes not known or ambiguous. In all species, the main region [in *Manduca*, the distal ileac plexus (67, 68)] of the tubule is shown.

The segregation of active cation transport to principal cells, and chloride and water flux to stellate cells, may confer selective advantages and could potentially extend to other insects. Stellate cells are more widely distributed than initially thought (17), and we have previously shown that fluorescently labeled kinin [the neuropeptide that stimulates the chloride conductance (5, 20)] marks stellate-like cells in most advanced endopterygote insects (51), suggesting an ancient and conserved role. To probe the route of water flux in insects without the benefits of *Drosophila* transgenics, we applied the dextran flux labeling technique to a panel of insects selected to represent the major exopterygote and endopterygote orders, so providing an initial view on the 2-cell model (Fig. 6). Among the endopterygote insects, dextran selectively labeled stellate-like cells of all insect orders except the beetles, where an extensive network was observed. Significantly, kinin genes are almost never found in Coleoptera (52), consistent with a lack of stellate cell specialization. In the more primitive exopterygotes, the story is more varied; although kinin had labeled the epithelium rather generally, this general pattern was seen in the locust, but not a cockroach. As a first approximation, therefore, the 2-cell model, that links chloride flux, kinin stimulation, and water flux, seems to have broad applicability across the higher insects.

### A Revised Model for a High-Flux Epithelium.

**Fig. 7.**
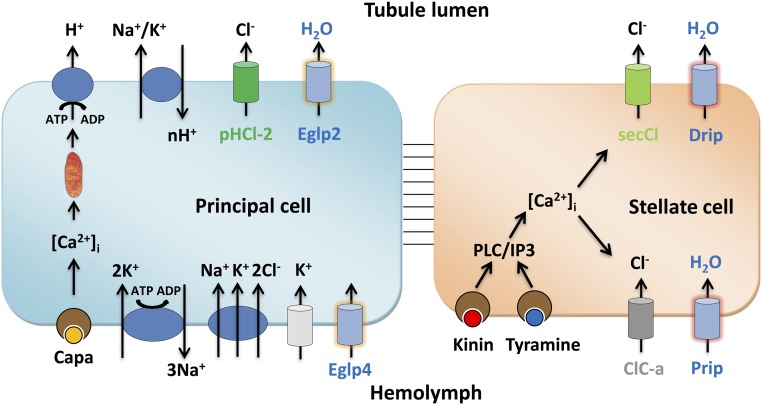
Model for tubule function. The mitochondria-rich principal cell is specialized for metabolically intensive cation and solute transport. The apical V-ATPase sets up a proton electrochemical gradient which drives net K^+^ secretion via NHA or NHE exchangers. Basolateral K^+^ entry is afforded by inward rectifier K^+^ channels, Na^+^,K^+^ ATPase and an Na^+^,K^+^,2Cl^−^ cotransport. The resulting lumen-positive potential drives a chloride shunt conductance, mainly via basolateral ClC-a and apical secCl channels in the stellate cell. The net transport of KCl drives osmotically obliged water, which is primarily via basolateral Prip and apical Drip in the stellate cells. In this way, the metabolically active principal cell is sheltered from the required high flux rates of water.

The tubule shows a remarkable ability to secrete primary urine at very high rates, and, together with other recent results, it is becoming clear that this success relies on the functional segregation of transport between different cell types (Fig. 7). The main, principal cell has long apical microvilli (45), each containing a mitochondrion (46) and loaded with proton-pumping V-ATPase, and is thought to drive an exchanger from the NHA family to produce a net K^+^ flux. Basolaterally, the infoldings contain high levels of Na^+^, K^+^-ATPase (10), inward rectifier K^+^ channels (11, 12), and Na^+^/K^+^/Cl^−^ cotransporters (16). This metabolically active cell is likely the route for excretion of a wide range of solutes via ABC transporters and other organic solute transporters, many of which are abundantly expressed in the tubule (26). The rarer stellate cells, by contrast, have shorter microvilli and fewer mitochondria, but are the gatekeepers for the hormone-stimulated chloride shunt conductance (through basolateral ClC-a and apical secCl) (23, 24), and now also for the passage of osmotically obliged water through basolateral Prip and apical Drip AQPs. The metabolically active principal cell is thus sheltered from these very high, and potentially disruptive, fluxes of water.

Given the severe consequences of unregulated fluid loss to a small terrestrial insect, it is not surprising that the tubule is under sophisticated neurohormonal control (53). Whereas cation pumping by the principal cells is under control of DH31 (54), DH44 (55), and Capa (56) neuropeptides, the stellate cells are independently controlled by the neuropeptide kinin (21, 57) and by the biogenic amine tyramine (19); both act indistinguishably through intracellular calcium (19, 58). The chloride shunt conductance is a known target of kinin and tyramine, as both rapidly collapse the lumen-positive potential (19, 20); however, it will be interesting to investigate whether one or both of these messengers have an independent action to regulate stellate cell AQPs, perhaps through phosphorylation or recruitment of AQPs to the plasma membrane.

This 2-cell model is likely to be widely applicably through the higher insects, the endopterygotes, which include flies, butterflies, and bees, in which a secondary cell type has been observed either directly (17), or by mapping an AQP (28), or by visualization with fluorescently labeled kinin (the hormone which regulates chloride flux) (51), or by otherwise mapping the kinin receptor (59, 60). However, a universal model is unlikely, as most members of one higher insect order (the Coleoptera) do not use kinin signaling (51), and, in the lower exopterygote insects, such as crickets, there is no evidence for specialized secondary cells. The next challenge will be to map out the generality of this 2-cell model, and its alternatives, across the tens of millions of species that make up the insects.

## Materials and Methods

### Informatics.

The MIP amino acid sequences were obtained from *Drosophila* gene database Flybase (flybase.org), and multiple sequence alignment was performed using Clustal Omega (https://www.ebi.ac.uk/Tools/msa/clustalo/). Phosphorylation sites were analyzed using GPS 3.0 algorithm (gps.biocuckoo.cn).

### *Drosophila* Stocks and Rearing.

Flies were reared at 22 °C, 45% relative humidity on a 12:12 photoperiod on standard *Drosophila* media. The lines (with original source) for this study were as follows: wild-type *D. melanogaster Canton-S* (Bloomington stock #1); *c724-*GAL4 (4) and *ClC-a*-GAL4 (23) (VDRC #202625) driver lines specific to stellate cells, and used interchangeably in this study; *CapaR*-GAL4 driver line specific to principal cells (61, 62); *UAS-Drip-Venus* (23); double-strand RNA (dsRNA) line directed against *Eglp2/CG17664* (VDRC #101847); *Eglp4/CG4019* (NiG-Fly stock #4019R-2); *Prip/CG7777* and *Drip/CG9023* (NiG-Fly stock #7777R-2 and #9023R-2, respectively).

### Generation of Transformants.

*UAS-Prip-Venus*, *UAS-Eglp2-Venus*, and *UAS-Eglp4-Venus* were generated by PCR amplifying the coding sequence of the respective *Drosophila* MIP genes using DreamTaq green PCR master mix (Thermo Fisher Scientific) and the primer pairs listed in *SI Appendix*, Table S1. Open reading frame amplicons were cloned into pENTR donor vector (Invitrogen) and transferred to pTWV destination vector (DGRC) using Gateway LR Clonase II Enzyme mix according to manufacturing instructions (Thermo Fisher Scientific). Sequence integrity was confirmed by Sanger sequencing (GATC Biotech), and transgenic lines were generated by using standard methods for P-element mediated germ-line transformation (BestGene).

### qRT-PCR.

For validation of tubule mRNA expression, qRT-PCR was performed using an ABI StepOnePlus Detection System (Applied Biosystems) with Brilliant III Ultra-Fast SYBR Green QPCR master mix (Agilent, UK) and the primer pairs listed in *SI Appendix*, Table S1. Data were normalized against the rpl32 standard and expressed as fold change compared to controls ±SEM (*n* = 3).

### Antibody Production and Immunohistochemistry.

Antigenic peptides were identified using Abdesigner software (63). Rabbit antipeptide antibodies were raised against the Drip epitope (CFKVRKGDDETDSYDF), Prip epitope (CNEASEKYRTHADERE), Eglp2 epitope (CSEVDETTMSTKRTSE), and Eglp4 epitope (CTSNEKLRQLEDVQLS) by Genosphere Biotechnologies.

Malpighian tubules from 7-d-old flies were dissected in Schneider’s medium (Thermo Fisher Scientific) and transferred to poly-l-lysine (Sigma-Aldrich)-covered 35-mm glass-bottomed dishes (MatTek Corporation) in PBS, fixed in 4% (wt/vol) paraformaldehyde in PBS for 30 min at room temperature, washed in PBT (PBS + 0.05% [vol/vol] Triton X-100), and then blocked in 10% (vol/vol) normal goat serum (Sigma-Aldrich) in PBT. IgG-purified rabbit anti-Drip/Prip/Eglp2/Eglp4 peptides (dilution 1:1,000) were used. Alexa Fluor 488/564-conjugated affinity-purified goat anti-rabbit antibodies (Thermo Fisher Scientific) were used at a concentration of 1:1,000 for visualization of the primary antiserum. Incubations in the primary and secondary antibodies were performed overnight at 4 °C. Tubules were incubated with the nuclear stain DAPI (1 μg⋅mL^−1^; Sigma-Aldrich) for 1 min and, in some cases, Rhodamine/Alexa-633 coupled phalloidin (1:100; Thermo Fisher Scientific) in PBT for a minimum of 30 min. Samples were washed repeatedly in PBS before being mounted in Vectashield (Vector Laboratories Inc.). Confocal images were taken using an LSM 880 inverted microscope (Zeiss) and processed with Zen black/blue software (Zeiss) and Adobe Photoshop/Illustrator CS 5.1.

### Western Blotting.

For each fly line, Malpighian tubules from >50 flies were dissected under Schneider’s *Drosophila* medium (Thermo Fisher Scientific) and were transferred to 100 μL of radioimmunoprecipitation assay buffer (150 mM NaCl, 10 mM Tris⋅HCl pH 7.5, 1 mM ethylenediaminetetraacetic acid, 1% Triton X-100, 0.1% [wt/v] sodium dodecyl sulfate) with 1 μL of protease inhibitor mixture (Sigma-Aldrich). Samples were homogenized using a Microson XL2000 sonicator (Misonix Inc.), and centrifuged (13,000 rpm) at 4 °C for 10 min. Protein concentrations were measured using the Bradford Protein Assay (Bio-Rad Technologies). Approximately 20 μg of protein from each sample was electrophoresed on a NuPage 4 to 12% Bis-Tris gel and blotted onto nitrocellulose membrane using the Novex system (Thermo Fisher Scientific). Blots were stained with Ponceau S and probed with IgG-purified rabbit anti-Drip/Prip/Eglp2/Eglp4 antibodies (1 μg⋅mL^−1^) and developed by electrochemiluminescence assay using enhanced chemiluminescence horseradish peroxidase-linked anti-rabbit IgG (1:2,000; Amersham Biosciences).

### Fluid Secretion.

Secretion assays were performed as described previously (2). Malpighian tubules from 7-d-old adult female flies were dissected under Schneider’s insect medium (Thermo Fisher Scientific) and isolated into 10-μL drops of a 1:1 mixture of Schneider’s medium: *Drosophila* saline, or a minimal *Drosophila* saline supplemented with glutamine (composition adapted from ref. 43). Intact tubules were left to secrete for ∼30 min before starting the experiment. Secretion rates were measured every 10 min; after 30 min of baseline readings, the diuretic peptides *Drosophila* kinin and capa-1 were added to 10^−7^ M, and secretion rates were measured for a further 30 min. Data are plotted as mean ± SEM (*n* > 7).

### Dextran Labeling.

Individuals were lightly anesthetized using either C0_2_ or ice, and their Malpighian tubules were dissected in Schneider’s *Drosophila* medium (Thermo Fisher Scientific). Dissected tissues were then preincubated for 10 to 20 min at room temperature in a solution of 1:1 Schneider’s:PBS with neuropeptides (e.g., Kinin, DH31, DH44) present at a concentration of 10^−7^ M (stimulated) or with no neuropeptides (nonstimulated). The dissected tissues were then transferred to fresh Schneider’s:PBS solution containing 0.2% dextrans (40 kDa or 70 kDa; Thermo Fisher Scientific) conjugated to a specified fluor, for 2 to 5 min at room temperature. Tissues were fixed for 10 min in 2% (wt/vol) paraformaldehyde, stained with 1 µg/mL DAPI (Sigma) for 2 min, transferred to poly-l-lysine (Sigma)-covered 35-mm glass-bottomed dishes (MatTek Corporation) in PBS, and imaged using a Zeiss LSM 880 confocal microscope (Zeiss).

### *Xenopus* Oocyte Assays.

The complementary DNA for *Drosophila* AQPs (Drip, Prip, Eglp2, Eglp4), human AQP4 and mefugu AQP8 were cloned into pGEMHE, a plasmid optimized for complementary RNA (cRNA) expression in *Xenopus laevis* oocytes. The cRNA synthesis, oocyte injections (10 ng/oocyte), and oocyte care were performed as previously (64). To ensure basic water channel activity before detailed analysis, oocytes expressing AQPs were placed in distilled water, and time for swelling and ultimately bursting was noted.

To calculate permeability to water (osmotic), glycerol, mannitol, and urea, we used a Zeiss Lumar, ZEN 2.0, and a 4-well perfusion chamber (1 to 1.5 mL) and acquired images every 5 s for 10 min. For osmotic water permeability, we diluted ND96 (200 mOsm) to 70 mOsm. Permeability for glycerol, mannitol, and urea was assessed by exposing oocytes to 200 mM solute (2 mM Hepes, pH 7.5) from ND96. Solutions were perfused into the chamber (full solution change in 20 s) beginning at 45 to 50 s into the experiment. To prevent contamination effects, that is, not returning to baseline volume, oocytes were only exposed to one osmotic or solute challenge. The experiments were repeated for each of the different substrates, and oocytes from at least 3 different donor *Xenopus* were used.

Water permeability (P_f_) was calculated as before (65, 66),

$$P_{f}=V_{0}\left[d\left(\frac{V}{V_{0}}\right)dt\right]/\left[\left(S\right)\left(\Delta Osm\right)\left(V_{w}\right)\right].$$ [1]

### Data Analysis.

Experiments were analyzed using a custom macro in FIJI. The image files were first converted to an 8-bit image and then made binary. The analyze particles function was used to measure the major (*a*) and minor (*b*) diameter of all 3 oocytes (3 µm per pixel). The third axis (*c*) for ellipsoid volume was calculated as the average of the major and minor axes. Ellipsoid surface area is calculated as

$${\text{SA}}_{\text{ellipsoid}}=4\pi *\sqrt[{p}]{\left[\frac{a^{p}b^{p}+a^{p}c^{p}+b^{p}c^{p}}{3}\right]},$$ [2]

where *p* ≈ 1.6075. This SA_ellipsoid_ is then multiplied by 8 to account for the SA convolutions of the oocyte. Finally, Permeability of solute (P_solute_) is calculated,

$${\text{P}}_{\text{solute}}\left(\text{cm}/\text{s}\right)=\Delta \left({\text{SA}}_{\text{ellipsoid}}/{\text{V}}_{\text{ellipsoid}}\right)/\Delta \text{t},$$ [3]

where Δt = each 20 s (4 point) interval after solute addition. P_solute_ (cm/s) was maximal by 120 s after change to 200 mM solute. We then used a 4-point rolling average 120 s after the test solute as the reported value P_solute_ (cm/s) for each oocyte. Numbers of injected oocytes used for each experiment are presented in *SI Appendix*, Table S2.

### Desiccation Stress Assay.

The 7-d-old male and female flies of specified genotype were anesthetized briefly with CO_2_ and placed in groups of 30 in empty vials (no food or water), and the open end of the tube was sealed with parafilm (Bemis). Flies were counted until 100% mortality was reached, and data were expressed as percent survival ± SEM (*n* = 3).

### Statistical Analysis.

GraphPad Prism 7.0 software (GraphPad Software Inc.) was used for statistical analysis and generation of graphs. For fluid secretion analysis, a 2-tailed Student’s *t* test, taking *P* = 0.05 as the critical value (for 2 independent groups: stimulated vs. basal), was used. For mRNA level quantification, one-way ANOVA followed by Tukey’s multiple comparisons of means with a significance level of *P <* 0.05 (for 3 independent groups) was used. For measurement of oocyte water and solutes permeability, we used an ANOVA analysis for each group, with a significance level of *P <* 0.05. For survival curves obtained in desiccation assays, significance was assessed by the log-rank (Mantel−Cox) test. Log-rank tests were conducted for each pairwise comparison.

### Data Availability Statement.

All original data are available from the corresponding author on request.

## Supplementary Material

Supplementary File
