# Supplementary material for: Specialized stellate cells offer a privileged route for rapid water flux in Drosophila renal tubule
Source: Proc Natl Acad Sci U S A. 2020 Jan 6;117(3):1779–87. doi: 10.1073/pnas.1915943117 (PMC6983416; doi:10.1073/pnas.1915943117)
Supplement: Supplementary File [file pnas.1915943117.sapp.pdf]

Supplementary Information

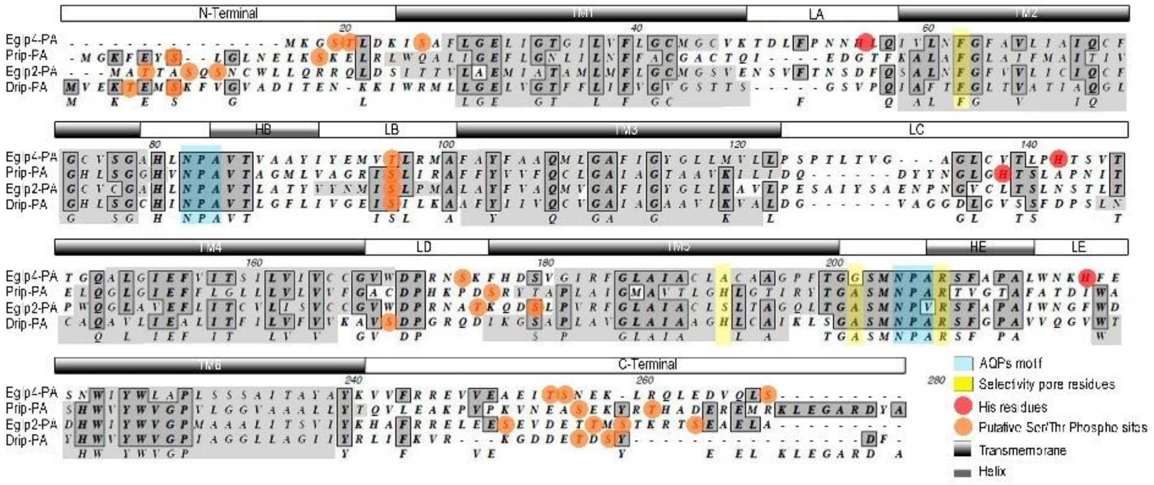

Fig. S1. Clustal Omega alignment of the amino acid sequences of *Drosophila melanogaster* aquaporins (Drip and Prip) and aquaglyceroporins (Eglp2 and Eglp4). MIP proteins consist of two tandem repeats, each of which has three membrane spanning α-helices and a pore forming loop with a signature Asp-Pro-Ala (NPA) motif (in blue). Highlighted in orange are the putative phosphorylation sites; in red are the Histidine residues involved in pH and divalent cations sensitivity; in yellow are the residues involved in pore selectivity.

www.pnas.org/cgi/doi/10.1073/pnas.1915943117

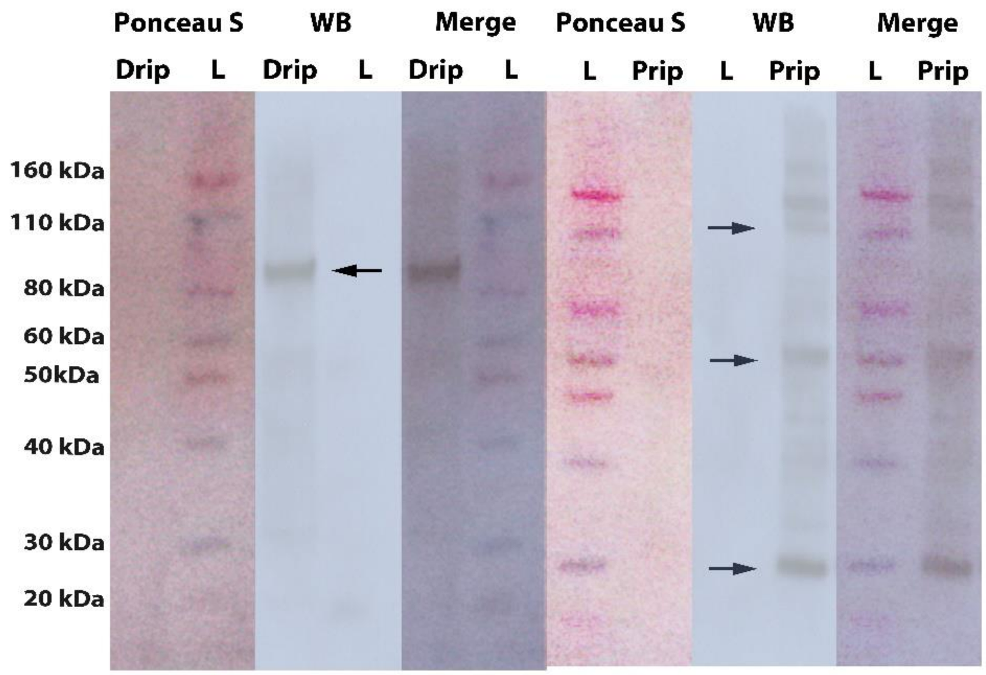

Fig. S2. Western blot of Malpighian tubules protein homogenates probed with Drip and Prip antibodies. Ponceau S staining and western blot of tubule protein samples of Drip and Prip antibodies. The predicted molecular mass of Drip and Prip monomer is 25.5 and 29 kDa respectively and specific bands corresponding to either monomer, and higher molecular weight bands corresponding to dimer and/or tetramer were detected (arrows).

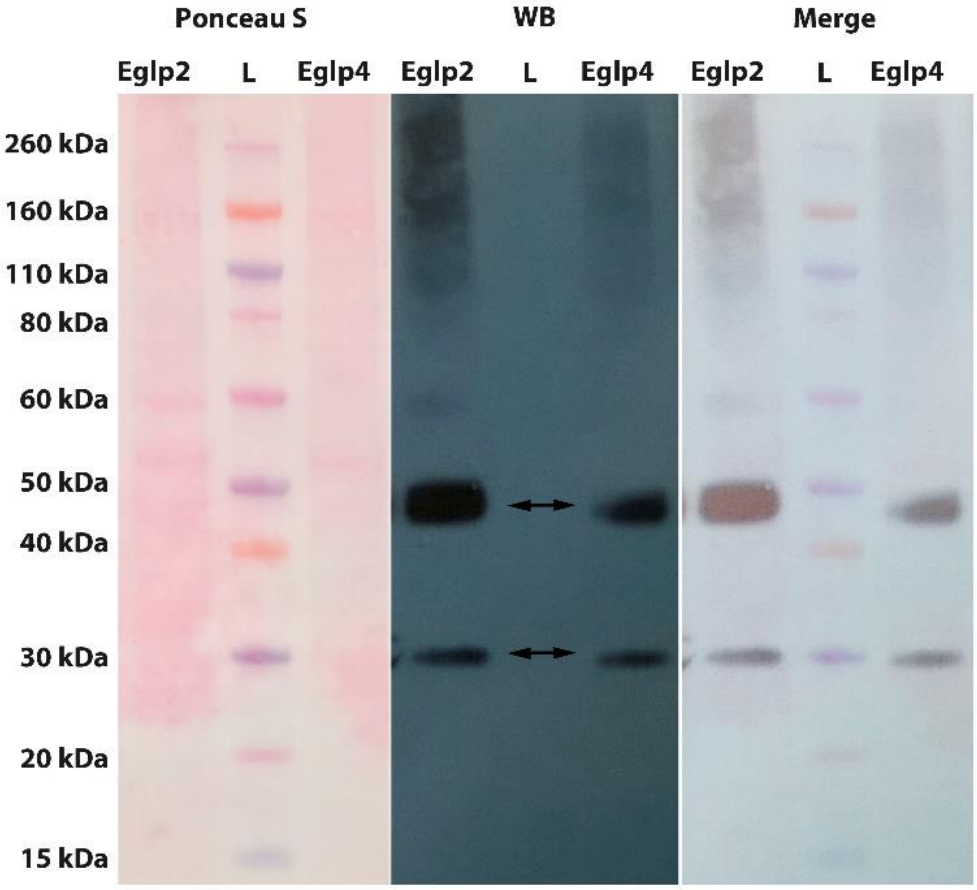

Fig. S3. Western blot of Malpighian tubules protein homogenates probed with Eglp2 and Eglp4 antibodies. Ponceau S staining and western blot of tubule protein samples of Eglp2 and Eglp4 antibodies. The predicted molecular mass of Eglp2 and Eglp4 monomer is 28.8 and 30.9 kDa respectively and specific bands corresponding to either monomer and dimer were detected (double arrows).

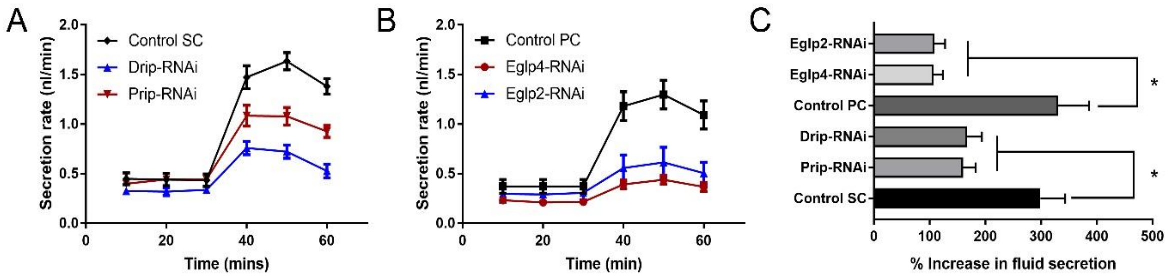

Fig. S4. Impact of cell-specific down-regulation of MIPs on fluid secretion. (*A*) Effects of Drip and Prip knockdowns in stellate cells and (*B*) Eglp2 and Eglp4 knockdowns in principal cells on stimulated fluid secretion by tubules in response to Capa-1 and Kinin at 10^{-7} M. (*C*) Data are expressed as percentage increase from basal fluid secretion compared to parental controls ± SEM (n = 6-12). *$P < 0.05$ (Student's *t*-test).

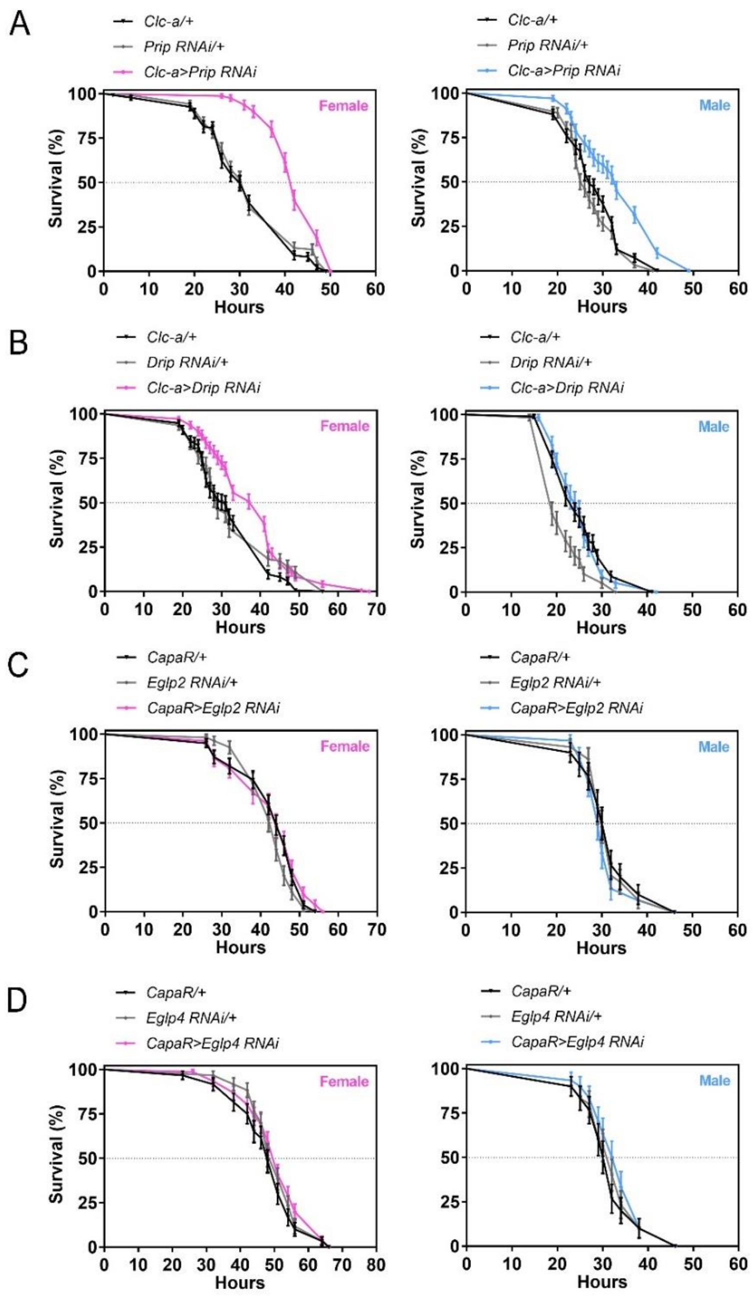

Fig. S5. Compromising the stellate route for water flux increases survival on desiccation. (*A*) Reduced Prip levels in stellate cells (*ClC-a*-GAL4>*UAS-Prip RNAi*) alter survival of desiccated flies. Desiccation resistance was significantly higher after knockdown of *Prip* in stellate cells compared to controls ($P < 0.001$ against both controls; Log rank test, Mantel-Cox). Median survival time (hours) are: female (*ClC-a>Prip* RNAi = 42, *ClC-a*/+ = 30, *Prip* RNAi/+ = 32); and male (*ClC-a>Prip* RNAi = 33, *ClC-a*/+ = 27, *Prip* RNAi/+ = 25.5). (*B*) Reduced Drip levels in stellate cells have a similar effect to Prip downregulation in female flies under desiccation conditions. Female (*ClC-a>Drip* RNAi = 41, *ClC-a*/+ = 31, *Drip* RNAi/+ = 29); and male (*ClC-a>Drip* RNAi = 24.5, *ClC-a*/+ = 24, *Drip* RNAi/+ = 19). Reduced levels of Eglp2 (*C*) and Eglp4 (*D*) specifically to principal cells does not alter survival of desiccated flies. Median survival time (hours) are: Eglp2 (female (*CapaR*>*Eglp2* RNAi = 44, *CapaR*/+ = 44, *Eglp2* RNAi/+ = 44); and male (*CapaR*>*Eglp2* RNAi = 29.5, *CapaR*/+ = 30, *Eglp2* RNAi/+ = 30)); Eglp4 (female (*CapaR*>*Eglp4* RNAi = 51, *CapaR*/+ = 48, *Eglp4* RNAi/+ = 51); and male (*CapaR*>*Eglp4* RNAi = 33, *CapaR*/+ = 31, *Eglp4* RNAi/+ = 32)).

Genes	Sequence (5’-3’)
**For qRT-PCR**	
**Eglp2 qPCR-F**	TGGACAGCATCACAACAGTTCTTG
**Eglp2 qPCR-R**	ACGAATCCGAAGTTCAGGGC
**Eglp4 qPCR-F**	TCGGAACGCAGTTGCTGTAAG
**Eglp4 qPCR-R**	TGGTTGTTGGGAAAGAGGTCC
**Drip qPCR**	TaqMan probe - Dm01792928_m1
**Prip qPCR**	TaqMan probe - Dm01792933_m1
**Overexpressor fly lines**	
**Eglp2 ORF-F**	CACCATGGCTACAACCGCAAG
**Eglp2 ORF-R**	GGCGAGCTCCGCCTCCGA
**Eglp4 ORF-F**	CACCATGAAGGGATCGACGCTGG
**Eglp4 ORF-R**	CGACAGCTGGACGTCCTCCA
**Drip ORF-F**	CACCATGGTCGAGAAAACAGAAAT
**Drip ORF-R**	GAAGTCGTACGAGTCGGTCTC
**Prip ORF-F**	CACCATGGGAAAATTCGAATACTCAC
**Prip ORF-R**	GGCGTAGTCACGGGCTCCCTC
***Xenopus* oocyte expression**	
**Eglp2 ORF-F**	ATGCGGCCGCCACCATGGCTACAACCGCAAG
**Eglp2 ORF-cmyc-R**	GCGGTACCTCACAGATCCTCTTCTGAGATGAGTTTTTGTTCGGC GAGCTCCGCCTCCGA
**Eglp4 ORF-F**	ATGCGGCCGCCACCATGAAGGGATCGACGCTGG
**Eglp4 ORF-cmyc-R**	GCGGTACCTCACAGATCCTCTTCTGAGATGAGTTTTTGTTCCGA CAGCTGGACGTCCTCC
**Drip ORF-F**	ATGCGGCCGCCACCATGGTCGAGAAAACAGAAAT
**Drip ORF-cmyc-R**	GCGGTACCTCACAGATCCTCTTCTGAGATGAGTTTTTGTTCGAA GTCGTACGAGTCGGTC
**Prip ORF-F**	ATGCGGCCGCCACCATGGGAAAATTCGAATACTCAC
**Prip ORF-cmyc-R**	GCGGTACCTCACAGATCCTCTTCTGAGATGAGTTTTTGTTCGGC GTAGTCACGGGCTCCC

Table S1. Primer sequences used in this study.

cRNA \ #oocytes	P(H_2O)	P(urea)	P(glycerol)	P(mannitol)
H_2O	26	31	18	9
hAQP4	14	14	13	2
Drip	14	16	16	8
Prip	10	8	12	6
Eglp2	16	14	15	12
Eglp4	12	10	10	6
fAQP8	14	11	13	2

Table S2. The table indicates the total number of oocytes for each MIP permeability experiment.
